# Systematic Review of Authentication and Authorization Advancements for the Internet of Things

**DOI:** 10.3390/s22041361

**Published:** 2022-02-10

**Authors:** Michal Trnka, Amr S. Abdelfattah, Aishwarya Shrestha, Michael Coffey, Tomas Cerny

**Affiliations:** 1 Department of Computer Science, Faculty of Electrical Engineering, Czech Technical University in Prague, 121 35 Prague, Czech Republic; trnkami1@fel.cvut.cz; 2 Computer Science, Baylor University, One Bear Place 97141, Waco, TX 76798, USA; amr_elsayed1@baylor.edu (A.S.A.); michael_coffey@baylor.edu (M.C.); 3 Computer Science, University of Wisconsin-Milwaukee, 3200 N Cramer St., Milwaukee, WI 53211, USA; shrest23@uwm.edu

**Keywords:** Internet of Things, authentication, authorization, identity management, survey, security

## Abstract

Technologies for the Internet of Things (IoT) are maturing, yet no common standards dictate their direction, leaving space for a plethora of research directions and opportunities. Among the most important IoT topics is security. When we design a robust system, it is important to know the available options for facing common tasks related to access control, authentication, and authorization. In this review, we systematically analyze 1622 peer-reviewed publications from October 2017 to December 2020 to find the taxonomy of security solutions. In addition, we assess and categorize current practices related to IoT security solutions, commonly involved technologies, and standards applied in recent research. This manuscript provides a practical road map to recent research, guiding the reader and providing an overview of recent research efforts.

## 1. Introduction

### 1.1. Background

Internet of Things (IoT) is an environment in which numerous heterogeneous and small devices interact and cooperate. However, the large number of cooperating devices raises numerous problems such as:

- With which participants can data be shared?
- Which participants can be interacted with?
- What is the best way to authenticate participants?
- What is the best way to detect a malicious node?
- What is the best way to introduce a new device into the network?
- What is the best way to retire the device, and when should this be done?

Devices in a network have different software versions, operating systems, and manufacturers and are often owned by different users. For this reason, IoT has become more complicated due to the heterogeneity of the nodes.

Building IoT based on the Internet makes it intrinsically inherent to the security problems from the Internet. In the initial stage of IoT development, security is typically not a significant concern to the users or stakeholders [1]. Security in this stage is often ignored [2] as the industry intends to push IoT to be commercialized as soon as possible. Nevertheless, with the rapid development of IoT, security issues have emerged due to the vulnerability of the nodes and the highly distributed and dynamic features of the underlying networks. Therefore, security is one of the most crucial challenges in the IoT system [3,4].

### 1.2. Motivation and Contribution

Numerous efforts have been made in IoT security research. Noor et al. published an IoT security survey [5] from a comprehensive viewpoint. While the study provides a wide range of perspectives for authentication and authorization, the survey is limited to the years 2016 to 2018. Our previous work on this topic includes another extensive survey [6] but is also limited to the years 2012 to 2017. The most recent overview of security challenges and their solutions is provided in [7]; however, it does not provide sufficient detail on authorization and authentication. Similarly, another study considers the detail of information security and privacy perspectives in IoT [8]. Continuous authentication methods are then elaborated in [9] through a survey that provides a great overview of the specialized perspective but not a general overview for authorization and authentication. Another prominent study [10] goes through industrial IoT security issues. An overview of the related studies is summarized in Table 1. These publications provide reasonable detail but are limited by years or focus on selected security perspectives or application areas, leaving gaps regarding the following three questions:

(1) What does current IoT authentication and authorization research look like?
(2) What are the common properties of IoT application-layer authentication and authorization solutions?
(3) How can a general researchers grasp the main trend of this area quickly?

This work is concerned with currently available IoT security solutions located at the application layer. Our contributions made in this work consist of two parts:

- We offer a useful roadmap of analyzed and distilled key information from recent 1622 peer-reviewed articles located at major academic sources. Unlike previous surveys and reviews focusing on the specific theme of IoT security, our work provides a blueprint to the general readers without much relevant background working in this area.
- Since the IoT application layer includes application-specific vulnerabilities such as authentication, authorization, identification, data management, and information privacy, we position this systematic review primarily concerning the taxonomy of security solutions, context-aware solutions adopted standards, and the distributed vs. centralized nature of given approaches and specific interactions.

The remainder of this manuscript is structured as follows. The goals of this manuscript are listed in Section 2. The literature identification is explained in Section 3. Resulting publications are categorized in Section 4. Respective research goals are elaborated in Section 5. Threats to validity are discussed in Section 6. Section 7 summarizes achieved goals. Finally, the conclusion of the survey is presented in Section 8.

## 2. Goals

This article presents the most recent findings and trends of IoT authentication, authorization, and identity management. Furthermore, it summarizes research efforts for the years 2017 to 2020, inclusive. This allows other researchers in the given domain to get an overview of the progress in the existing research, learn ideas from other publications, shape research into a broader context, and determine the overall direction of the current scientific efforts.

The benefits are not limited only to the scientific audience. The survey lists the primary research on which future production-ready applications (commercial and open-source) will be based. They will serve a significantly larger community, including users with no technical or scientific background.

This survey aims to answer the following research questions:

RQ1 What is the taxonomy of security solutions?
RQ2 Which topologies, communication types, and perspectives are most dominant in the authentication and authorization IoT research?
RQ3 What are the applicability domains and requirements of identified solutions?

The first goal is to group the research into various categories based on similar properties. The second goal explores architecture decisions that affect (de)centralization of the solution, suitability for machine-to-machine (M2M) and user-to-machine (U2M) communication, and usage of context-aware elements. The third goal evaluates whether the solution is generally applicable or is best for a specific domain, or whether specialized tools are needed to implement it (physical access tokens, cameras, etc.).

## 3. Literature Identification

This systematic review utilizes the following indexing sites to identify evidence: IEEE Xplore, ACM Digital Library (ACM DL), Web of Science (WoS), SpringerLink, and ScienceDirect. Previously published studies [5,6] have proven relevant in the search for scientific evidence and relevant to the review scope but are now dated. We approach this study intending to avoid wheel reinvention. Thus, instead of considering the overall time interval, this study provides an update including publications through the end of 2020. We reuse the same general query from our previous survey review relevancy [6]. It contains an already established, formulated, and tested query, which matches the scope discovered through manual searching, to considered indexing sites. However, we apply current time constraints to integrate recent literature by a complete year. Such an approach warrants continuity across the current study and the previous one.

The considered search query is devised of two distinct parts: the items to include and the items to exclude. We describe target terms and keywords that we expect to find in our results to begin our query. The first keyword that we specify is “Internet of Things” or “IoT”, followed by the term “Security”. These keywords are obvious due to the survey we are conducting; however, there is an enormous amount of research on IoT Security, so we must continue to narrow our search to produce useful results from our query. To constrain our results further, we specify that we only want to include papers with the terms “Authentication”, “Authorization”, “Access Control”, or “Identity” (short for identity management). After specifying what we wanted to find from our query, we added to the query what we wanted to exclude from our results. We excluded papers that discuss security at a low level in the network stack to narrow our search. To accomplish this, we discarded any papers containing the keywords “Network”, “Hardware”, “RFID”, and “protocol”. Furthermore, we are not focusing this survey on “Cryptography”, so we removed any papers with this keyword as well. To end our query, we ensured that our results did not include any surveys by removing all papers with the keywords “Survey” and “Study” in their title.

Due to the differences in the searching procedure found at each site, we turned the query into appropriate forms for each indexer. To promote similarity between indexer results, we manipulated the general query for each individual indexer just enough to get the desired result. We did not want to use queries that were exceedingly different. The general query, along with the individual queries used for each indexer, is listed in Table 2. Performing these queries returned 1622 results, as detailed in Table 3.

To select relevant publications, we established inclusions and exclusion criteria, which we detail below. These criteria are applied to all 1622 results. We proceeded as follows: in the first round of elimination (prefiltering), we considered publication abstract, title, and keyword assessment. When it passed the inclusion and exclusion criteria, we included the publication in the next stage in the next stage. There, we read the full text of the candidate publication and decided whether it was in the scope based on the ability to decode answers for the questions that we raised in this systematic review. The reduction process with relevant publication numbers is detailed in Table 3.

### 3.1. Inclusion and Exclusion Criteria

The inclusion criteria for the publications can be summarized with the following list:

- Published between October 2017 and 2020 (both inclusive).
- Indexed by either IEEE Xplore, ACM DL, WoS SCIE, SpringerLink, or ScienceDirect.
- Relates to authentication, authorization, identity management, or access control for IoT. In particular, we considered whether the publication proposed a solution to considered topics.

To narrow down the scope, we have also formed exclusion criteria that are applied to the included articles:

- Not written in English.
- Duplicate publication.
- Published before October 2017 (considering our previous survey time scope [6]).
- Less than four pages.
- Could not determine the technical objective (mainly because of poor English).
- Not in the scope of the application layer, i.e., focused on security on the lower level of the network stack.
- Survey or opinion publication without explicit technical contribution.
- Utilized blockchain technology.

Blockchain is excluded from the result not because it does not fall into the scope but rather because of its high prevalence. There were over one hundred articles focused on blockchain technologies for the IoT. To detail the perspective, our previous survey [6] contained only two blockchain articles. This illustrates the massive increase in blockchain-related research. Thus, we do not discuss the differences between blockchain technologies in the scope of a general review due to their similarities from a high-level perspective.

### 3.2. Searched and Filtered Results

After the queries were run over all indexing services, we were presented with a set of 1622 publications considering inclusion and exclusion criteria from Section 3.1. We were then able to eliminate one duplicate publication found by the WoS indexer. Finally, we read the abstract of each article and eliminated any publications that did not fit within the scope of this survey, giving us 214 prefiltered candidates.

**Figure 1:**
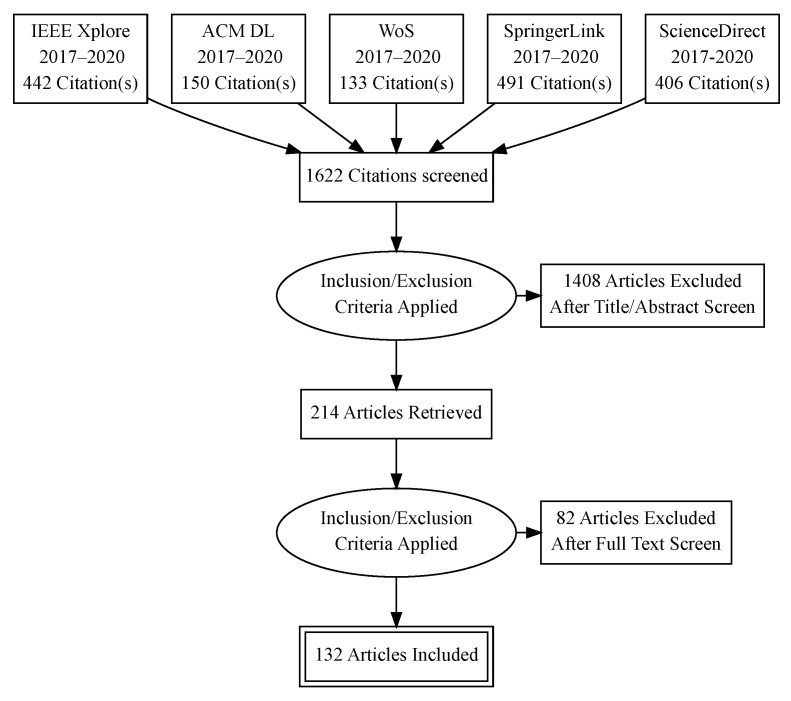
Illustration of the survey process-flow inclusion and exclusion of articles.

Upon completion of the filtering process, we read through the remaining publications, categorized them based on the criteria discussed in this survey, and performed property coding detailed in Section 3.3. During this read-through, we were able to remove more articles that at first looked as though they fit our scope but upon further examination were proven unrelated. The complete statistics of publications found, prefiltered, and included for every indexing site can be seen in Table 3. This shows that the indexer, IEEE Xplore, returned 442 total results originally, 90 articles remained after prefiltering, and 76 articles were declared relevant. ACM DL returned 150 results originally; 43 articles remained after prefiltering, and 28 articles were declared relevant. WoS returned 133 results originally; 56 articles remained after prefiltering, and 16 articles were declared relevant. SpringerLink returned 491 results originally; 6 articles remained after prefiltering, and 2 were declared relevant. Finally, ScienceDirect returned 406 articles originally; 19 articles remained after prefiltering, and 10 were declared relevant. The summation of these indexers showed 1622 articles were originally returned; 214 articles remained after prefiltering, and 132 were declared relevant. The survey process-flow is illustrated in Figure 1.

### 3.3. Property Coding

Each publication that passed through inclusion and exclusion criteria was read full-text with the intent to extract information relevant to this study. If we could not extract the information, we excluded the publication.

We assessed each publication’s metadata (i.e., years, conferences, authors, etc.). In the full text, we targeted the target domain, motivations, and goals to categorize the metadata. We determined whether the particular publication topic applies a specific approach in the application layer if it is a context-aware approach for addressed and architectural properties, such as whether the solution tends to be centralized or decentralized. We assessed whether any specific constraints were assumed for the solution and devices and if a special device (i.e., external one) is needed for the considered approach. We also identified where the schema applied to both user-to-machine and machine-to-machine interactions. We compiled all considered publications into a large roster detailed in the taxonomy section based on this coding scheme.

## 4. Taxonomy and Trends

Categorizing the filtered publications into specific groups is one of the main goals of this survey. This categorization is done with three different taxonomy models; they are described in the following subsections:

1. Years-based Taxonomy.
2. Goals-based Taxonomy.
3. Automation-based Taxonomy.
4. The three-year perspective trends.

### 4.1. Years Based Taxonomy

**Figure 2:**
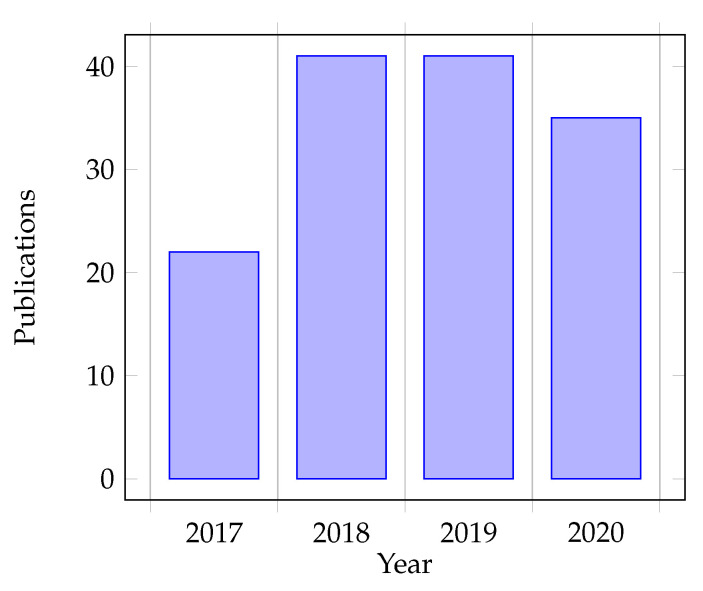
Number of publications per year.

This graph projects data in the period of October 2017 till December 2020. Since early 2017, published papers have already been included in the previous survey [6]; the graph starts from October 2017. As represented in Figure 2, the values of the graph show a fair increase in the number of papers regarding this research scope since the last survey we conducted, such that it varies in the range of 30 to 50 papers per year. However, it is noted that the number of papers slightly decreased in 2020, probably because of the appearance of the COVID-19 pandemic that affected most industries and fields then.

### 4.2. Goals-based Taxonomy

Assigning the filtered papers into specific and predefined categories (as detailed in Section 3.3) is one of the main directions of this article. Therefore, four categories are explored to satisfy the research goals and to answer the research questions mentioned in Section 2. Accordingly, the characteristics of papers are fully surveyed to be classified into the following categories:

1. *Context-awareness (yes/no)*: the ability of a system to gather information about its environment at any given time and adapt behaviors accordingly.
2. *Centralized vs. decentralized network topology (centralized/decentralized/both or N/A)*: the solution topology could require either centralization, decentralization, or combination between such elements.
3. *Communication model (M2M/U2M/both or N/A)*: the different communication methods in terms of the machine-to-machine (M2M) or user-to-machine (U2M), which strictly require some user input information.
4. *Existing vs. new method (existing, new, extension)*: the novelty of the method. It is unusual for solutions to be novel as a whole. It is common to reuse existing technology in novel ways.

These categories are considered common and valuable for most IoT approaches. Therefore, they are individually described in the upcoming sections. One contribution of this survey is the large property coding described in Section 3.3, and it is shared through Table 4 and Table 5.

### 4.3. Automation Based Taxonomy

To further broaden our categorization, we utilized automated algorithms. In particular, we used the automated algorithms that produced the most common categories among all of the relevant publications. For this process we used *pdftotxt* [143] for transforming the PDF documents into plain searchable text. Then, the *RAKE* [144] algorithm was used for keyword extraction. After that, the extracted keywords were grouped together into 10 major categories. Note that categories are not exclusive in such a process, and one publication can be a member of multiple categories at the same time.

For 12 publications [11,12,13,14,15,16,17,18,19,20,21,22], no categorization was detected automatically due to generic keywords extracted (i.e., "devices" or "Internet"), which are not closely related to one of the major categories. For these papers, we extracted their keywords manually.

**Figure 3:**
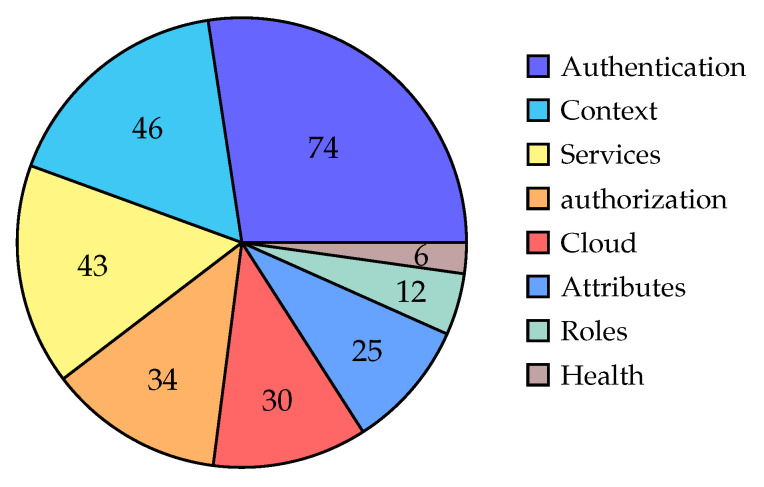
Number of articles in each category.

This taxonomy process produces the following eight categories, are shown in Figure 3 with their included number of publications:

1. *Authentication*: [12,23,24,25,26,27,28,29,30,31,32,33,34,35,36,37,38,39,40,41,42,43,44,45,46,47,48,49,50,51,52,53,54,55,56,57,58,59,60,61,62,63,64,65,66,67,68,69,70,71,72,73,74,75,76,77,78,79,80,81,82,83,84,85,86,87,88,89,90,91,92,93,94,95]
2. *Context*: [11,14,18,22,23,29,32,35,36,37,39,41,42,44,48,49,53,57,64,69,70,74,76,80,90,92,96,97,98,99,100,101,102,103,104,105,106,107,108,109,110,111,112,113,114,115]
3. *Services*: [26,33,35,37,38,39,40,41,44,46,48,52,57,65,66,69,72,81,84,85,86,87,93,97,98,103,106,107,111,113,116,117,118,119,120,121,122,123,124,125,126,127,128]
4. *Authorization*: [13,15,16,17,18,19,20,21,32,33,35,37,38,39,40,48,49,53,58,59,64,71,74,79,81,91,95,99,103,111,122,126,129,130]
5. *Cloud*: [24,29,37,38,39,41,56,57,59,60,63,70,71,75,83,85,86,87,94,95,99,103,117,131,132,133,134,135,136,137]
6. *Attributes*: [23,29,32,33,37,49,53,65,74,75,97,99,100,103,104,108,110,111,114,124,125,131,138,139,140]
7. *Roles*: [32,33,37,65,71,97,100,106,111,120,141,142]
8. *Health*: [85,100,104,106,118,135]

The resulting categories are expected for security IoT research. The major one is *Authentication*, followed by *Context* and *Services*. The first one is closely related to the nature of the IoT solutions with their access to the context, and the second one illustrates distributed nature of those solutions. The top three categories are followed in fairly closed order by *Authorization*, *Cloud*, and *Attributes*, and the two least populous are *Roles* and *Health*. We have included those two only as illustrations for comparison with the previous survey from 2017 [6]. Figure 3 illustrates the categories with respect to the number of included papers.

There are interesting observations, such as that Attribute-Based Access Control (ABAC) [145] has become increasingly popular for security. This is due to its higher flexibility and ability to better describe complex rules. Vice-versa, the Role-Based Access Control (RBAC) [146], is slowly losing its popularity. One other interesting observation is that there are very few healthcare applications. In our last survey [6] from 2017, 14% of the papers were concerned about healthcare. In contrast, now it is only 4.4%.

### 4.4. The Three-Year Perspective Trends

Compared to our previous survey [6], we can observe trends. We can constantly see high interest in the authentication (51% before vs. 55% now). The second most populous category now is context, which has a share of 35% versus 23% in 3 years ago. Our perspective shows that IoT security research is moving towards solutions that can capitalize on one of the main IoT advantages (inherent access to context). Services have experienced a slight loss in popularity (37% vs. 32%). The authorization research has dropped significantly from 46% to 25%. However, we attribute that to the fact that we have skipped all research related to blockchain solutions in this study. Moreover, in our experience, blockchain is a promising technology to share security rules, and therefore most of the omitted papers would fall into this category. The cloud category does not exhibit any significant popularity changes (19% vs. 22%). Finally, the ABAC, as mentioned above, is getting more popular, with an increase from 14% to 19%. Roles are still a minor topic. The most surprising category is healthcare. There is a notable drop in healthcare solutions. In 2017, 14% of related publications were concerned about healthcare. In contrast, now it is only 4.4%. What was a notable research topic three years ago was identity management and tokens. However, these were not identified for the current publications.

## 5. Details on Goal-Based Taxonomy Perspectives

Goals-based taxonomy is summarized by Table 4 and Table 5. We discuss the statistics in the following subsections.

### 5.1. Context-Awareness

Context-awareness in IoT is the ability of a system to gather information about its environment by detecting context entities using various methods, such as collecting data via sensors, smartphones, tablets, wearable devices, smart bands, cameras, microphones, GPS devices, and user input. The collected information can be turned into higher-level knowledge and is useful in various applications. Utilizing this functionality, numerous objects in the environment are monitored, notify the consumer of potentially dangerous situations, provide the ability to communicate with trusted devices, and address eventual accidents. These abilities allow for increased safety, efficiency, and economic benefit for those environments. In this subsection, we first provide the papers that utilize contextual information to achieve security in an IoT environment using authentication and access control techniques, and then we present papers that could meaningfully avail information in various domains and perspectives.

Authenticating a user is paramount when it comes to security. When implemented in conjunction with password-based authentication methods, context-aware authentication systems append an additional security layer. They can replace the conventional authentication methods. For example, in the paper [98], to strengthen authentication security, the author has presented a dynamic authentication model for accessing smart home devices by utilizing traditional credentials with context-aware information. Context-aware authentication is an important characteristic of smart homes. The goal of context-aware authentication systems in smart homes is to provide security services that maximize the user’s comfort and safety while minimizing the user’s explicit interaction with the environment [46,49,147]. For instance, ref. [35,48,66] utilizes location-based information in authentication framework for smart environment.

The growth of IoT technology presents excellent opportunities but also produces many new challenges related to authentication in IoT devices. Using passwords or pre-defined keys has drawbacks that limit the use of different IoT applications. Thus, authenticating users on password mechanisms is not always feasible. To overcome this issue, some papers focus on different authentication methodologies. For example, one paper implements JSON Web Token (JWT) [148], which is an open standard that uses encoded JSON objects as a payload while transmitting information between two parties [75,119], and Two Microphone Authentication [105], which uses the audio and network channel to authenticate commands. It provides an additional security check against maliciously injected commands.

IoT device authentication is fundamental to ensure the identity of connected devices can be trusted. Alongside authentication, Access Control (AC) provides selective restriction of access to services and data, or for performing a certain operation on a resource, service, or connected object. There are various paradigms of access control mechanisms, which are shifted from fixed desktop to dynamic context-aware environments. Mainstream approaches in access control systems include RBAC [146] and ABAC [145]. For example, to mitigate malicious attacks in IoT environments, the paper [87] uses the Role-Based Reputed Access Control method and achieves device security by tracking the activities of the device based on location. With ABAC, access decisions are made based on attributes (characteristics) about the subject. While RBAC covers broad access [65,111], ABAC can control access on a more detailed level. Several researchers have developed ABAC models that support context-based access control [20,32,104,113,125].

Different types of dynamic context information bring new challenges to access control systems. To improve the classic access control techniques, Alianea and Adda published an extension to the ABAC in the form of the High-Order Attribute-Based Access Control model (HoBAC) [114]. This new model makes it possible to implement IoT AC policies based on hierarchies of entities (objects, subjects, and environment attributes) built using aggregation operations on the attributes of existing entities. Furthermore, ref. [103] presents additional functionality in which the model not only considers system-wide attributes-based security policies but also takes into account the individual user privacy preferences for allowing or denying services. Additionally, utilization of context information can also be performed in Operation-Based Access (grouping is performed on the basis of operations instead of roles) [97], Event-Based Access Control (only authorized device can send data and initiate the events) [13], Capability-Based Access Control (CBAC) [130], and Hybrid Access Control Model [100] (a combination of RBAC, ABAC, and CBAC, employing attributes, roles, and capabilities). Moreover, in order to improve access control mechanisms, contextual information can also be taken into consideration at the time of trust value verification [108,112].

A context-aware system uses heterogeneous data sources to adapt and provide services to the user according to his needs, his localization, or his interaction with the environment. This results in the ubiquitous source of context data in mobile devices that can provide different services in different contexts—where context is strongly related to a device’s location. Due to this, most initial research in context-aware computing focused on location-aware systems. For example, context-based information is utilized from the MAC address of devices that support information such as the owner name or location for user authentication [92]. However, context is more than just location. Biometric data as addressed in [22,92,102] are also considered “contextual” by definition. Contextual data help to obtain the background information and can be used to frame what you know in a larger picture. Moreover, the authors of [28] utilized the contextual data using Radio Frequency Identification (RFID) technology.

In summary, 58 context-aware solutions have been proposed in systems, middleware, applications, techniques, and models [15,16,18,20,21,22,23,24,26,32,35,36,44,46,48,49,52,53,59,60,63,65,66,72,73,74,75,76,77,86,87,90,91,92,95,97,98,100,101,102,103,104,105,107,108,111,112,113,114,120,121,124,125,129,133,137,139,142]. The particular works that address context-awareness are shared in Table 4 and Table 5. They can be used to address different challenges in IoT. The results in these papers clearly show the importance of context-awareness in the IoT paradigm.

### 5.2. Distributed vs. Centralized Network Topology

With IoT systems, we typically expect to follow the decentralized nature of solutions. However, authentication and authorization are sometimes designed with centralism in mind. This leads us to two strategies of security solutions: distributed and centralized.

The *centralized approach* has benefits related to global governance and simplicity. It is easy to control and enforce identical policies across the ecosystem from a single focal point. Moreover, this model allows for migration between non-IoT-based software and that which is IoT-based. However, the drawbacks of this approach include the potential lack of scalability and creating a system bottleneck; this implies potential issues with resilience and a single point of failure. Centralized approaches often consider a component in the middle [95]. This approach seems natural for smart homes [59,77,93,103,130] and cars where the scale does not introduce an issue. However, as apparent from Table 4 and Table 5, this is not not always the case [69,98].

In contrast, the *distributed approach* addresses concerns related to resilience and scalability by not relying on a central node for processing. The distributed solution makes individual nodes more responsible for their logic, which limits coupling. However, this approach adds a layer of complexity to the system’s synchronization, maintenance, and auditing. It also introduces a new problem, whether devices can be trusted.

Distributed Secure Multi-Party Computation (SMPC) nodes [120] were used to make policy decisions for authentication of devices. These learn device behavior and limits using a distributed registry and assemble a decentralized decision based on the honesty of a device.

In order to produce a scalable, decentralized public key distribution scheme, [55] called for a decentralized, permissionless Public Key Infrastructure (PKI) running on a blockchain. First, it ensures that the public keys belong to the real device and owner without involving a Trusted Third Party (TTP). Second, it considers an authentication flow to define the process for an entity to grant approval to access a resource.

A History-Based Capability System (HCAP) [129] regulates the order in which permissions are exercised in a distributed authorization environment. HCAP capabilities carry sequencing constraints in the form of security automata. An HCAP works well as a building block suite for centralized policy administration and decentralized policy enforcement.

The rule-attribute-based access control model proposed in [19] targets a distributed environment. It is based on using digitally signed documents or certificates that convey identity, authorization, and attributes.

A data protection framework introduced in [16] has a set of constraints for policy construction. It proposed an access-control framework (policy-based language) to govern the security operation of distributed data in dynamic IoT networks.

In [132], smart meters mutually authenticate each other with a service provider to establish a session key for secret communication.

A security framework for edge-computing [118] has been connected to healthcare systems. It utilizes multi-factor access control and ownership transfer mechanism to create an authentication system. Furthermore, scalability is achieved by employing a distributed approach for clustering techniques that analyze and aggregate voluminous data acquired from heterogeneous devices individually before it transits to the cloud.

Unfortunately, the ability to distinguish a solution between these two categories is not always possible. Some solutions can work with both centralized and distributed environments, causing a blur in the categorization. Due to this, we have split up these categories further by introducing the subcategories: strictly centralized, strictly distributed, both, and not applicable.

Identity management through a centralized server is essential for certain practices due to the added difficulty of securing distributed operations. This added security may be a result of IoT in a specific domain [46], or it might just be derived from the methods or technologies employed [33,109,111,112,134]. The authors of [123] proposed a self-sovereign identity offered by distributed ledger technology to provide a secure, decentralized, and persistent identity for IoT devices. This allows a device identity, along with all its relationships, to be securely managed throughout its entire lifecycle.

However, some proposals decide to take a distributed approach by necessity. ABAC systems [32,33,97,100,103,104,111,114,124,125,140] rely on peer devices for entity attribute confirmation, which also requires a distributed architecture to function.

To summarize from the identified publications, as shown in Table 4 and Table 5, we identified 47 centralized approaches [11,18,20,22,23,26,32,33,38,39,42,43,46,48,49,51,53,59,60,65,67,68,70,71,75,76,77,82,88,91,93,94,95,101,102,103,109,111,112,122,125,127,130,132,133,134,137], 56 distributed ones [12,13,16,21,24,27,30,31,35,37,40,44,47,52,54,55,56,58,62,63,64,69,72,73,79,81,83,85,87,89,92,96,97,98,100,104,105,106,108,110,113,114,115,116,118,120,121,123,124,129,131,135,136,139,141,142], and 6 approaches that use both [17,28,66,99,126,138]. Finally, 23 publications were not relevant or did not specify the results.

### 5.3. Communication Model

The communication model can be seen in the perspectives of machine-to-machine (M2M) or user-to-machine (U2M). U2M strictly requires some user input information. IoT interaction may, similar to other distributed systems, consider stimuli from users or other autonomous parts of the system. The subcategories to encompass all articles involve M2M, U2M, and both.

*U2M communication* centers around user actors. Thus, we need to authenticate users, either in a conventional way or sometimes through unconventional means such as biological information [32,46,66,77,102,104,117] or even forms such as a user’s mental state [39].

For *M2M communication*, there is no restriction that U2M systems possess (user stimuli/intervention); however, the abilities of these systems are often limited to the initial programming of users upon setup. M2M enables devices on the network to exchange information and perform actions without the manual assistance of humans. This fits IoT as the common use case is to tap into sensor data and transmit it to a target system for processing or further escalation of automated actions. There are times when a user wants the responsibility of maintaining the security of an IoT system [66,120,140], and this is where M2M becomes valuable. Among examples, monitoring, supply chain management, and smart homes are all great fits for IoT solutions.

Authentication and authorization for M2M use cases are designed in [79], which specifically discusses one M2M security architecture, OAuth 2.0 framework, and Mobius. A more specific use case can be shown through a video surveillance system [115]. This system enables the active (automatic) monitoring of the controlled areas as it allows for the detection and the pre-alarm of abnormal events in real-time. A Diffie–Hellman-inspired protocol was used to allow two smart cameras to share a secret image. A tunneling framework was provided to protect the M2M communications established between the cameras using their fingerprints as an authentication factor and a secret image they share as a cipher key. A password-based authentication scheme for M2M Networks in [25] was achieved using hash invocations and symmetric key encryption. The scheme is suitable for environmental sensors, which are limited in resources (computation, storage, energy, etc.) Furthermore, a novel security model approach in [65] introduced security-related attributes combined with privilege management infrastructure to overcome known drawbacks in machine-to-machine communication such as poor extensibility, lacking use-case-related authorization schemas, and weak separation between information and authorization model.

There may be instances where either a U2M or M2M model exists in a researched technology. For these instances, a M2M tool can be transformed to work with a U2M model [81], or the tool can exist in both models [33,38,56,74,111].

To summarize, we identified 57 U2M approaches [11,14,18,20,22,24,26,29,31,32,35,39,40,42,44,46,48,50,51,59,60,62,64,68,70,73,75,76,77,78,80,82,83,85,92,93,94,98,100,102,104,105,106,107,112,118,119,122,126,128,132,133,135,136,137,139,141], 31 M2M ones [13,21,25,27,28,43,54,55,65,66,79,87,88,89,95,96,99,101,103,109,110,113,114,115,120,125,127,129,130,138,140], and 30 approaches where both were used [12,16,17,23,30,33,34,38,45,47,49,52,53,56,63,67,71,72,74,81,91,97,108,111,116,121,124,131,134,142]. Finally, 14 publications were not relevant or did not specify the model. The majority of centralized topologies involved U2M. For distributed topology, the distribution was slightly in advance of U2M. There was no impact of the communication model for the context-aware solutions.

### 5.4. Existing versus New Methods

Here, we consider the method novelty. It is common to build on existing solutions and extends them, but some researchers propose novel alternatives.

In this taxonomy, current publications on IoT security can roughly be divided into three categories: applying existing methods, extending them to better suit the IoT specifications, and building new methods.

Studies that adapted or applied existing technologies and methods from other security domains to the IoT environment often considered extensions to ABAC and RBAC [32,33,97,100,103,104,111,114,124,125,140]. While RBAC is a method of restricting network access based on the roles of individual users within an enterprise, ABAC is an authentication and authorization model under the identity management umbrella that uses attributes rather than roles to grant users access.

Other interesting technologies in this taxonomy include OAuth 2.0 [81,134] and the Fuzzy logic system [42,109]. OAuth 2.0 is an authorization protocol that is used by online applications to gain access to resources hosted by other online applications. The Fuzzy logic system is an attempt to imitate how people think through computation. This allows reasoning to be considered regarding a problem, as opposed to an approach with basic evaluation. Three works focused on the use of JSON Web Token (JWT) [148] for different authentication techniques [75,119,124]. JWT is similar to regular web tokens, but it contains a set of claims to transmit information between two entities.

Studies that focus on novel ideas make use of very diverse methods to achieve unique results. One proposal [26] introduces the security framework, named SODA, to centralize security policy and service management for IoT environments, acting as an intermediate device that all devices are connected to, which allows all security concerns to be routed through it. Other approaches based on physical authentication discuss the use of brainwaves for authentication by considering the familiarity between users and certain images [39], and two methods use an accelerometer to measure a person’s gait and authenticate the user based on this metric [60,76].

In summary, there are various brand-new proposals with novel ideas in 14 publications [13,31,33,36,37,41,46,61,65,86,90,93,109,127] that have great potential to address the IoT security issue from the perspectives of scalability, maintainability, and flexibility. However, it is still difficult to predict which ideas might be adopted widely. Although a significant amount of research is focused on adoption of existing technologies with 63 of identified publications [15,16,20,21,22,23,26,28,30,32,35,39,40,43,45,49,50,51,55,57,60,62,63,66,67,69,71,72,73,76,78,82,84,92,94,95,96,98,99,100,101,102,103,104,105,107,112,113,116,118,120,121,123,125,126,129,133,135,137,138,139,140,141], there were 55 publications related to extensions [11,12,14,17,18,19,24,25,27,29,34,38,42,44,47,48,52,53,54,56,58,59,64,68,70,74,75,77,79,80,81,83,85,87,88,89,91,97,106,108,110,111,114,115,117,119,122,124,128,130,131,132,134,136,142].

Extensions were more common for context-unaware works. There was no significant impact from topologies or communication models.

### 5.5. Domains and Constraints Used in Research of Security Solutions

The publications we assessed considered security solutions in general and specific domains. Table 4 and Table 5 indicate the specifics for each publication. Overall, nearly half of the publications considered solutions applicable to any domain, which we classify as general. Among specific domains were mentioned IoT platforms, smart homes, healthcare, fog computing, wearable, surveillance, ATM, smart city, and cars.

Often, the security solutions considered constrained devices or even a specialized or external device such as smartwatches and other wearable technology, which we highlight in Table 4 and Table 5 as well.

Most approaches did not require special devices. However, a large number required special devices such as biometric sensors, wearables, cameras, security devices, mobile devices/smartphones, and RFID and sensors.

## 6. Threats to Validity

It is usual for systematic reviews, mapping studies, and surveys to suffer from several threats to validity that need to be addressed. We have discovered multiple threats to mention. In this context, we discuss the validity threats from the perspective of Wohlin’s taxonomy [149]. In particular, four potential threats are considered: construct validity, internal validity, external validity, and conclusions validity.

The construct validity is meant to consider the research questions within the investigated area. Our queries are motivated by a previously performed study, which dated results. The primary terms were combined with secondary terms and exclusion parts to execute this study. All used terms are commonly recognized in the community and domain of this work, and all are suitable to be used as search strings. A possible threat of omitting relevant research from our review was addressed by experimenting with several other search strings identifying related work. Still, this study could miss relevant work, although given threads would be slightly impacted. Moreover, selected major research databases were considered but not all. The analyzed sample only considered peer-reviewed articles published by journals or conferences to ensure the objectivity and reliability of the information sources. It did not include reprints of the papers submitted to or accepted in journals and conferences published by arXiv.org, researchgate.net, or individual personal pages. These reprints might contain novel ideas, methods, and new challenges relevant to the scope of analyzed papers. Furthermore, our article queries were limited to the abstracts of the articles, so we could have missed relevant work with poorly stated abstracts.

Internal validity involves methods to study and analyze data (e.g., the types of bias involved). One potential threat is related to inclusion and exclusion, a process that included metadata, abstracts, and possibly full-text assessments; this could be further affected by our bias when performing the filtering. Multiple authors performed this, with primary authors assigned to a particular indexer and secondary authors to spot-checking. Apart from the filtering process, we performed question coding, leading to improper interpretation of results. We addressed the above by assigning distinct indexers to different researchers, with spot-check validation by others on sample publications. Our goal-based taxonomy is a result of our discussions of interpreted results and represents our view on the identified literature. We also performed automated keyword extraction meant to address potential bias threats.

External validity is related to knowledge generalization. This survey interprets and categorizes works we gathered from established scientific channels, and our observations related to IoT. We could have missed related work; however, we aimed to minimize the impact possibly resulting from the presented trends and their generalization given through the diversity of scientific channels.

The conclusions result from several brainstorming sessions independently settled by all authors. To address the validity of the conclusions, we involved multiple authors in this study, with all of them discussing the outcomes in the context of extracted and synthesized information.

## 7. Answers to Research Questions

In this paper, we raised multiple Research Questions (RQ) addressed throughout the previous sections. Next, we provided more concise answers to the RQs with back-references to the particular section content.

***RQ1*** *What is the taxonomy of security solutions?*

This question is answered through three taxonomy models of distinctions by publication year, goals, and categorization through extracted keywords in Section 4. In particular, Figure 2 details the publication year taxonomy, with the year 2019 being the most active year. The keyword taxonomy is highlighted in Figure 3, giving the proportions of research focus on authentication, context, services, authorization, cloud, attributes, roles, and health.

***RQ2*** *What topologies, communication types, and perspectives are most dominant in the authentication and authorization IoT research?*

We have identified that IoT solutions become more context-aware when considering the perspectives of past and present. There is no conclusion to be made whether centralized or distributed models dominate; both are used with the moderate majority for distributed models as described in Section 5.2. The user-to-machine communication model has a significantly greater scientific interest than machine-to-machine models, although a hybrid model accommodating both is also considered as Section 5.3 stated in detail. The great majority of research works are on established methods.

***RQ3*** *What are the applicability domains and requirements of identified solutions?*

We addressed this question through full-text analysis, which resulted in comprehensive answers available in Table 4 and Table 5. A summary of the results is shown in Section 5.5.

## 8. Conclusions

This systematic review provides a practical overview of recent IoT authentication and authorization advancements. Using a systematic literature review approach, it assessed 1622 peer-reviewed publications to find evidence to provide security solution taxonomy and discuss recent efforts from other related perspectives. The provided details show that common practices and models are applied for authentication and authorization. Context-awareness can be a beneficial companion to aid authentication. While most security solutions are distributed, still a significant proportion are centralized. Research directions are further fragmented by communication from the central perspective to users or devices, for which we provide a practical road map to existing works.

## Figures and Tables

**Table 1:** Overview of related work.

Publication	Published	Summary
Noor et al. [5]	2019	A comprehensive overview of authentication and authorization research for years between 2016 and 2018.
Trnka et al. [6]	2018	Mapping study for authentication and authorization articles from 2012 to 2017.
Chanal et al. [8]	2020	Survey providing an overview of architectures, privacy and research challenges, and differences of solutions between domains.
Milovlaskaya et al. [7]	2019	Great overview of IoT back-end security issues, general hardware, and application security, along with a summary of IoT security management and security standards.
Al-Naji et al. [9]	2020	Focused survey on continuous authentication methods.
Tange et al. [10]	2020	Focused survey on industrial IoT security issues.

**Table 2:** Queries used for the search.

Indexer	Query
General query	(“Internet of Things” OR “IoT”) AND “Security” AND (“Authentication” OR “Authorization” OR “Identity” OR “Access control”) AND NOT (“Network” OR “Hardware” OR “RFID” OR “Protocol” OR “Cryptography” OR “Survey” OR “Study”)
IEEE Xplore	((“Abstract”: “Internet of Things” OR “Abstract”: “IoT”) AND (“Abstract”: “Authentication” OR “Abstract”: “Authorization” OR documentAbstract: “Identity” OR “Abstract”: “Access Control”) AND “Index Terms”: “Security” AND NOT(“Index Terms”: “Network” OR documentAbstract: “Hardware” OR “Abstract”: “Cryptography” OR “Abstract”: “Protocol” OR “Document Title”: “Survey” OR “Abstract”: “RFID” OR “Document Title”: "Study"))
ACM DL	Abstract: (IoT “Internet of Things”) AND Abstract: (“Authentication” OR “Authorization” OR “Identity” OR “Access Control”) AND Title: (-study -Survey) AND Abstract: (-Hardware -rfid -Cryptography) AND Keyword: (-Hardware -Physical -Network)
WoS SCIE	TI = (Internet of Things OR IoT) AND TS = (Authentication OR Authorization OR Identity OR Access Control) NOT TS = (Hardware OR Cryptography OR Protocol OR RFID OR Physical OR Network) NOT TS = (Survey OR Study) AND TS = Security
SpringerLink	‘(Authentication OR Authorization OR Identity OR “Access Control”) + title (“Internet of Things” OR IoT)’
ScienceDirect	(“Internet of Things” OR “IoT”) AND (“Authentication” OR “Authorization” OR “Identity” OR “Access control”) AND NOT (“Hardware” OR “Cryptography”)

**Table 3:** Number of articles processed in the survey.

Indexer	Results	Prefiltered	Relevant
IEEE Xplore	442	90	76
ACM DL	150	43	28
WoS	133	56	16
SpringerLink	491	6	2
ScienceDirect	406	19	10
Total	1622	214	132

**Table 4:** Selected paper categorization part 1/2.

References	Context Aware?	Topology (Centr./Distr.)	Communication Model	Existing vs. New	Domains	Constrained/ Unconstrained Devices	Required Special or External Devices
Ibrahim et al. [11]	N	C	U2M	Extension	Smart Home	C	Biometric
Baruah et al. [12]	N	D	Both	Extension	Industrial IoT Devices	C	Sensor, Router
Zulkipli et al. [13]	N	D	M2M	New	General	N/A	-
Chen et al. [14]	N	N/A	U2M	Extension	General	C	Biometrics ECG
Kashmar et al. [15]	Y	N/A	N/A	Existing	General	N/A	-
Karimibiuki et al. [16]	Y	D	Both	Existing	General	U	-
Chen et al. [17]	N	Both	Both	Extension	General	U	-
Olazabal et al. [18]	Y	C	U2M	Extension	Biometrics	U	-
Terkawi et al. [19]	N	N/A	N/A	Extension	General	N/A	-
Hoang et al. [20]	Y	C	U2M	Existing	General	N/A	-
Cattermole et al. [21]	Y	D	M2M	Existing	General	N/A	-
Mathew et al. [22]	Y	C	U2M	Existing	Home security	C	Biometrics
Jain et al. [23]	Y	C	Both	Existing	Automated Attendance System	U	Camera
Guo et al. [24]	Y	D	U2M	Extension	Fog Computing authentication	C	-
Renuka et al. [25]	N	N/A	M2M	Extension	IoT Environment	N/A	-
Kim et al. [26]	Y	C	U2M	Existing	General	U	-
Felde et al. [27]	N	D	M2M	Extension	Dynamic groups	U	-
Mahbub et al. [28]	N	Both	M2M	Existing	General	C	RFID
Heydari et al. [29]	N	N/A	U2M	Extension	Fog Computing	N/A	-
Ning et al. [30]	N	D	Both	Existing	General	U	-
Leung et al. [31]	N	D	U2M	New	General	C	Smart Watch
Bilgen et al. [32]	Y	C	U2M	Existing	General	U	-
Oh et al. [33]	N	C	Both	New	IoT Platforms	U	-
Dammak et al. [34]	N	N/A	Both	Extension	General	C	-
Nespoli et al. [35]	Y	D	U2M	Existing	IoT Environments	U	-
Rothe et al. [36]	Y	N/A	N/A	New	General	N/A	-
Ouaddaha et al. [37]	N	D	N/A	New	General	N/A	-
Yan et al. [38]	N	C	Both	Extension	Home security	C	Smart device (Door Lock), Smartphone
Chiu et al. [39]	N	C	U2M	Existing	Wearable Devices	C	Wearable brainwave headsets
Phoka et al. [40]	N	D	U2M	Existing	Security door	C	IR Sensor
Heydaria et al. [41]	N	N/A	N/A	New	General	N/A	-
Malarvizhi et al. [42]	N	C	U2M	Extension	Multi-bio authentication	C	Biometric scanners
Sharif et al. [43]	N	C	M2M	Existing	Road Construction	N/A	-
Ashibani et al. [44]	Y	D	U2M	Extension	Smart Home	C	Sensor
Ulz et al. [45]	N	N/A	Both	Existing	General	U	-
Gebrie et al. [46]	Y	C	U2M	New	Healthcare and Smart Home	C	Biometrics
Wang et al. [47]	N	D	Both	Extension	General	U	-
Nespoli et al. [48]	Y	C	U2M	Extension	IoT Platforms	C	Security devices, Sensor
Ghosh et al. [49]	Y	C	Both	Existing	Home IoT platform or Web service	C	Security devices
Gad et al. [50]	N	N/A	U2M	Existing	General	C	-
Mbarek et al. [51]	N	C	U2M	Existing	Smart Home	C	-
Hasan et al. [52]	Y	D	Both	Extension	General	C	Maxim DS2411
Arfaoui et al. [53]	Y	C	Both	Extension	General	U	-
Murphy et al. [54]	N	D	M2M	Extension	General	C	Accelerometers
Durand et al. [55]	N	D	M2M	Existing	General	N/A	-
Pallavi et al. [56]	N	D	Both	Extension	Fog computing	C	Sensor
Saadeh et al. [57]	N	N/A	N/A	Existing	General	N/A	-
Carnley et al. [58]	N	D	N/A	Extension	Smartphone Devices	U	-
Chifora et al. [59]	Y	C	U2M	Extension	Smart Home	U	-
Batool et al. [60]	Y	C	U2M	Existing	Healthcare	C	Electrocardiogram (ECG)
Gamundani et al. [61]	N	N/A	N/A	New	Smart Home	N/A	-
Chauhan et al. [62]	N	D	U2M	Existing	General	C	Smartphone, Smartwatch, Raspberry Pi
Dabbagh et al. [63]	Y	D	Both	Existing	All Wireless devices	U	Biometrics
Ali et al. [64]	N	D	U2M	Extension	Healthcare	U	-
Wallis et al. [65]	Y	C	M2M	New	General	U	-
Krašovec et al. [66]	Y	Both	M2M	Existing	General	C	Sensors
Yang et al. [67]	N	C	Both	Existing	Healthcare	C	Sensor
Sahoo et al. [68]	N	C	U2M	Extension	General	U	-
Zhu et al. [69]	N	D	N/A	Existing	Smart Home	C	-
Das et al. [70]	N	C	U2M	Extension	Industrial Internet of Things	C	Biometric sensor
R. Khan [71]	N	C	Both	Existing	General	U	-
Chien [72]	Y	D	Both	Existing	General	U	-
Aski et al. [73]	Y	D	U2M	Existing	Healthcare	U	Raspberry pi
Alkhresheh et al. [74]	Y	N/A	Both	Extension	IoT Platforms	C	Raspberry Pi
Ethelbert et al. [75]	Y	C	U2M	Extension	Cloud SaaS Applications	U	-
Sun et al. [76]	Y	C	U2M	Existing	Wearable Devices	C	Accelerometer

**Table 5:** Selected paper categorization part 2/2.

References	Context Aware?	Topology (Centr./Distr.)	Communication Model	Existing vs. New	Domains	Constrained/ Unconstrained Devices	Required Special or External Devices
Shayan et al. [77]	Y	C	U2M	Extension	Smart Home	C	Smart phone, Biometrics
Elganzoury et al. [78]	N	N/A	U2M	Existing	Mobile banking	U	-
Oh et al. [79]	N	D	M2M	Extension	General	C	-
Zhou et al. [80]	N	N/A	U2M	Extension	General	U	Brainwave Sensor
Oh et al. [81]	N	D	Both	Extension	IoT platforms	C	Sensor
Belk et al. [82]	N	C	U2M	Existing	General	U	-
Hassan et al. [83]	N	D	U2M	Extension	Wearable Devices	C	Smart phone
Kaliya et al. [84]	N	N/A	N/A	Existing	General	U	-
Wazid et al. [85]	N	D	U2M	Extension	Medicine validity detection	C	-
Shah et al. [86]	Y	N/A	N/A	New	General	N/A	-
Amoon et al. [87]	Y	D	M2M	Extension	Any access-control	U	-
Yazdanpanah et al. [88]	N	C	M2M	Extension	Wireless Sensor Networks	C	Sensor
Barbareschi et al. [89]	N	D	M2M	Extension	Computing Fog	C	-
Loske et al. [90]	Y	N/A	N/A	New	General	N/A	-
Shahzad et al. [91]	Y	C	Both	Extension	General	U	-
Rattanalerdnusorn et al. [92]	Y	D	U2M	Existing	IoT Environments	U	-
Prathibha et al. [93]	N	C	U2M	New	Smart Home	U	Biometrics
Whaiduzzaman et al. [94]	N	C	U2M	Existing	Fog IoT Environment	U	-
Liu et al. [95]	Y	C	M2M	Existing	Smartphone-centric	C	Smartphone
El Kalam et al. [96]	N	D	M2M	Existing	General	N/A	-
Genç et al. [97]	Y	D	Both	Extension	Smart device	U	-
Ashibani et al. [98]	Y	D	U2M	Existing	Smart Home	U	-
Bhatt et al. [99]	N	Both	M2M	Existing	General	N/A	-
Pal et al. [100]	Y	D	U2M	Existing	Healthcare (only Smartphone Device)	C	-
Miettinen et al. [101]	Y	C	M2M	Existing	General	N/A	-
Lu et al. [102]	Y	C	U2M	Existing	General	C	Biometrics
Gupta et al. [103]	Y	C	M2M	Existing	Cars, Vehicles	C	Cars Location Tools
Salama et al. [104]	Y	D	U2M	Existing	Healthcare	C	-
Blue et al. [105]	Y	D	U2M	Existing	General	C	Microphones
Islam et al. [106]	N	D	U2M	Extension	Healthcare	U	-
Srinivas et al. [107]	Y	N/A	U2M	Existing	Industrial Internet of Things	C	Smartcard, Biometrics
Pal et al. [108]	Y	D	Both	Extension	General	U	-
Atlamab et al. [109]	N	C	M2M	New	General	U	-
Khalil et al. [110]	N	D	M2M	Extension	IoT Environments	U	-
Djilali et al. [111]	Y	C	Both	Extension	IoT Platforms	U	-
Van hamme et al. [112]	Y	C	U2M	Existing	General	N/A	-
Schuster et al. [113]	Y	D	M2M	Existing	General	N/A	-
Alianea et al. [114]	Y	D	M2M	Extension	Any access-control	U	-
Nakouri et al. [115]	N	D	M2M	Extension	Video Surveillance Systems	U	Camera, Fingerprint sensor
Ranaweera et al. [116]	N	D	Both	Existing	Multi-access Edge Computing platform	N/A	-
Selvarani et al. [117]	N	N/A	N/A	Extension	General	N/A	-
Aski et al. [118]	N	D	U2M	Existing	Healthcare	U	Biometrics
Ahmed et al. [119]	N	N/A	U2M	Extension	General	U	-
Lupascu et al. [120]	Y	D	M2M	Existing	Industrial IoT Devices	C	IoT device/Sensor
Krishnan et al. [121]	Y	D	Both	Existing	Controlled IoT device	C	Blockchain, Sensor
Jonnada et al. [122]	N	C	U2M	Extension	Remote Collaboration Systems	U	-
Gebresilassie et al. [123]	N	D	N/A	Existing	General	N/A	-
Martinez et al. [124]	Y	D	Both	Extension	Smart city	C	Smartphone, Smart meter
Colombo et al. [125]	Y	C	M2M	Existing	General	N/A	-
Rech et al. [126]	N	Both	U2M	Existing	Cross-Domain Service	C	Bluetooth
Lee et al. [127]	N	C	M2M	New	General	N/A	-
S. Hazra [128]	N	N/A	U2M	Extension	ATM service	C	Biometrics
Tandon et al. [129]	Y	D	M2M	Existing	General	U	-
Shieng et al. [130]	N	C	M2M	Extension	Smart Home	C	-
Xiong et al. [131]	N	D	Both	Extension	IoT Cloud Storage	U	-
Wu et al. [132]	N	C	U2M	Extension	Distributed Cloud Computing	U	-
Han et al. [133]	Y	C	U2M	Existing	General	U	-
Fremantle et al. [134]	N	C	Both	Extension	IoT Platforms	U	-
Daoud et al. [135]	N	D	U2M	Existing	Healthcare cloud environment	C	Sensor, ECG
Cui et al. [136]	N	D	U2M	Extension	General	U	-
Vorakulpipat et al. [137]	Y	C	U2M	Existing	Card reader, finger print reader	C	Cards
Li [138]	N	Both	M2M	Existing	General	U	-
Gur et al. [139]	Y	D	U2M	Existing	IoT Platforms	C	IHG
Gong et al. [140]	N	N/A	M2M	Existing	Smart city	C	Sensor
Gwak et al. [141]	N	D	U2M	Existing	General	U	-
Chen [142]	Y	D	Both	Extension	Security	C	Sensors

## Data Availability

Not applicable.

## Abbreviations

The following abbreviations are used in this manuscript:

IoT	Internet of things
RQ	Research question
WoS	Web of Science
SCIE	Science Citation Index Expanded
ACM DL	Association for Computing Machinery digital library
M2M	Machine to machine
U2M	User to machine
ABAC	Attribute-based access control
RBAC	Role-based access control
JWT	JSON web token
GPS	Global positioning system
PC	Personal computer
CBAC	Capability-based access control
RFID	Radio-frequency identification
